# Haematinic and Hepatoprotective Properties of *Telfairia occidentalis* Fruit Mesocarp on Phenylhydrazine-Induced Anaemia in Experimental Rats

**DOI:** 10.1155/2023/8838481

**Published:** 2023-10-09

**Authors:** Ada Francesca Nneoyi-Egbe, Eridiong Onyenweaku, Andyno Akpanukoh, Patricia Ebai

**Affiliations:** ^1^Department of Biochemistry, Faculty of Basic Medical Sciences, University of Calabar, Calabar, Nigeria; ^2^Department of Human Nutrition and Dietetics, Faculty of Basic Medical Sciences, University of Calabar, Calabar, Nigeria; ^3^University of Douala, Higher Teacher Training College for Technical Education, Douala, Cameroon

## Abstract

The level and potential of iron contained in fluted pumpkin (*Telfairia occidentalis*) has been exploited as a blood tonic; however, the potentials of some other parts of the plant are unknown. The effect of *T. occidentalis* fruit mesocarp (aqueous extract) on phenylhydrazine (PHZ)-induced anaemia in experimental rats was investigated in a bid to determine its curative properties and potential in reversing haemolytic anaemia and protection of liver health. The LD_50_ of the fruit extract was determined using Lorke's method for the determination of acute toxicity. The study involved oral administration of varying doses of the extract to different groups of rats which were monitored for 24 hours. The test sample did not show any signs of toxicity at doses of 5000 mg/kg b.wt, which is the highest possible recommended dose for toxicity testing. For the evaluation of the effects of the fruit extract on haematological indices and biochemical enzyme markers in anaemic rats, 30 matured albino Wistar rats were used. The rats were divided into five groups of six rats each. Group 1 consisted of normal rats (control group), Group 2 consisted of anaemic untreated rats, and Group 3 consisted of anaemic rats treated with the standard drug Astymin, while Groups 4 and 5 were made up of anaemic rats given the extract at doses of 600 mg/kg b.wt and 1000 mg/kg b.wt, respectively. The fruit extract failed to show any significant effect in improving the haemoglobin (Hb), alanine aminotransferase (ALT), aspartate aminotransferase (AST), and alkaline phosphate (ALP) levels in anaemic rats but rather may have contributed to a reduction in Hb levels and an unhealthy increase in serum enzyme levels. This is indicative of the apparent inability of the aqueous extract of the *T. occidentalis* fruit mesocarp to reverse PHZ-induced haemolytic anaemia and may suggest a possible detrimental effect of high doses of the extract over a prolonged period.

## 1. Introduction

Anaemia is characterized by the reduction in red blood cell (RBC) count, haemoglobin (Hb) content, and packed cell volume (PCV). Generally, reduction in all three occurs due to decreased production of RBCs, increased destruction of RBCs, and excess loss of blood from the body [1]. Studies reveal that anaemia may be caused by inherited disorders or environmental factors such as nutritional problems, infections, and exposure to certain drugs or toxins [2]. Typically, gradual development of anaemia produces vague symptoms such as fatigue, weakness, shortness of breath, or a poor ability to exercise; however, abrupt development has more severe symptoms such as confusion, dizziness, loss of consciousness, or increased thirst. Furthermore, although anaemia is often characterized by paleness, it is only in significant cases [2].

Although the three main types of anaemia are identified primarily due to either blood loss, decreased RBC production, or increased RBC breakdown, anaemia can also be classified based on the morphological and etiological classification of anaemia. This includes macrocytic normochromic anaemia, macrocytic hypochromic anaemia, and nutrition deficiency anaemia (like iron deficiency) [2]. According to various studies, treatment largely depends on the cause of the anaemia. A typical example is in the case of iron deficiency anaemia, where a replacement iron therapy is usually prescribed. The two categories of iron supplements are those containing the ferrous form of iron and those containing the ferric form of iron, with the former being the better absorbed of the two [3]. Although iron supplements are widely used in treating cases of anaemia, certain chemical substances are capable of inducing or bringing about the onset of anaemia in otherwise living organisms, one of such being phenylhydrazine (PHZ).

**Figure 1:**
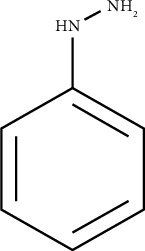
Structure of phenylhydrazine [4].

Research has shown that PHZ is a particularly potent redox-active drug capable of inducing haemolytic anaemia, even in individuals without erythrocytic enzyme deficiencies [3]. This is a result of haemolysis caused by interaction with sulfhydryl groups, inhibition of various enzymes, immune mechanisms, and the fragmentation of erythrocytes as they pass through the platelet-fibrin mesh or by unknown or poorly defined mechanisms. Yeshoda's study [4] induced anaemia in rats following a single PHZ intraperitoneal administration at a dose of 20 mg/kg b.wt (aqueous solution). In this study, erythrocyte concentration lowered to about 50% and haemoglobin level to about 60% of normal values in the course of 4 days. This is consistent with various data that reveal that PHZ induces anaemia via the destruction of red blood cells by oxidation stress and many joint changes at cellular levels. It is worth mentioning, however, that although various studies highlight the ill effects of PHZ on RBCs, few studies have tested the effects of other substances (like medicinal plants) on PHZ-induced anaemia.  Figure 1 shows the structure of phenylhydrazine.

The medicinal application of plants is common in Africa as the efficient use of these plants has been well established [5]. Ancient Egyptian medicine of 1000 BC was known to have used garlic, opium, castor oil, coriander, mint, indigo, and other herbs for medicinal purposes. In the seventh century AD, the Slavic people used *Rosmarinus officinalis, Ocimum basilicum,* and *Allium sativum* as a remedy against several injurious insects (such as lice, fleas, moths, mosquitoes, and spiders) [6]. However, in more recent years, the bark of willow trees which contains large amounts of salicylic acid (which is the active metabolite of aspirin) has been used as an effective pain reliever and fever reducer [7].

*Telfairia occidentalis* is a tropical vine grown in West Africa as a leaf vegetable and for its edible seeds. It is commonly known as fluted gourd or fluted pumpkin and is grown or planted in the forest zone of West and Central Africa, most frequently in the Benin Republic, Nigeria, and Cameroon [8]. In a study by Anthony and Ojeifo [9], the result of phytochemical screening of *T. occidentalis* leaf, root, fruit mesocarp, and stem showed impressive amounts of flavonoids, alkaloids, saponins, terpenoids, tannins, steroids, and glycosides, with the roots having more of these phytochemicals than other plant parts. The fruit mesocarp also showed high levels of tannins and steroids. Generally, although the level and potential of iron contained in fluted pumpkin has been exploited as a blood tonic which can be administered to weak patients [10], there is still uncertainty, however, about the benefits and potentials of other parts of the plant. In addition, the increase in malnutrition globally has prompted several studies on the impact of different foods on human health and well-being. Depending on their nutritional value, certain foods are considered healthier than others [11]. According to FAO [12], the composition/quality of various foods depends on several factors: species, breeds, cultivars, ecological factors, postharvest handling, preservation, and storage techniques. Consequently, this study seeks to investigate the haematinic and hepatoprotective properties of fluted pumpkin mesocarp (aqueous extract) on PHZ-induced anaemia in experimental rats.

## 2. Materials and Methods

### 2.1. Sample Collection and Preparation

**Figure 2:**
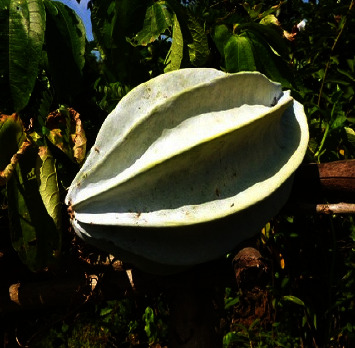
Picture of *Telfairia occidentalis* plant (personal communication).

**Figure 3:**
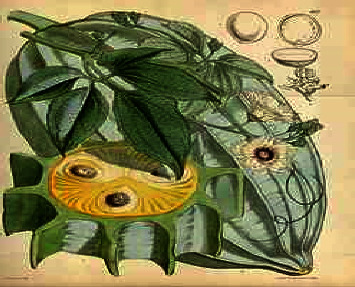
Diagram of the cross-sectional area of *T. occidentalis*, showing the exposed fruit mesocarp (yellow part), leaves, flowers, and seeds [13].

Fresh matured pods of *Telfairia occidentalis* fruit were obtained from Marian Market, Calabar Municipal, Cross River State. The plants were taken to the Botany Department, University of Calabar, for identification and confirmation. Figures 2 and 3 show diagrams of *T. occidentalis.*

The protocol for plant preparation and extraction was based on the work by Olorunfemi et al. [14]. The fruits were boiled for 45 minutes after which it was opened and the mesocarp scrapped off. The mesocarp was then blended into a thick paste using an electric blender. The resultant paste was macerated with distilled water for 72 hours to obtain the aqueous extract. The extract was sieved with a cheese cloth and subsequently with a filter paper in order to obtain a fine extract. The filtrate was then evaporated in a water bath. The brownish thick paste obtained was stored in a refrigerator for laboratory analyses.

### 2.2. Experimental Design

#### 2.2.1. Acute Oral Toxicity Testing

Twelve experimental mice of 330.6 ± 2.24 g body weight were purchased from the animal house, Department of Biochemistry, University of Calabar, Cross River State. The animals were housed in transparent wire-gauzed plastic cages to acclimatize for 5 days. The rats were fed with pelletized poultry grower's mesh feed and tap water *ad libitum.* The extract was administered once, orally after the animals were left to fast for 24 hours. Lorke's method (a new method for determining acute toxicity) as described by Enegide et al. [15] was used to determine the acute oral toxicity of aqueous extract of *Telfairia occidentalis* fruit mesocarp using twelve mice in all. This method consisted of different stages (Table 1) with the result of each stage determining whether to proceed or terminate and go for the confirmatory test. A confirmatory or confidence test was used to validate any test sample which showed signs of toxicity or caused mortality.

The test sample was administered 50 mg/kg, 200 mg/kg, 400 mg/kg, and 800 mg/kg body weight in stage 1 which involved four animals: one animal each per dose; stage 2 involved three animals, each received 1000 mg/kg, 1500 mg/kg, and 2000 mg/kg body weight, respectively. Lastly, stage 3 also had three animals, and each received 3000 mg/kg, 4000 mg/kg, and 5000 mg/kg body weight, respectively. At each stage, animals were observed for any signs of toxicity or mortality for 24 hours at an interval of 10 minutes and, thereafter, after every 1-2 hours. If the test sample did not show any sign of toxicity or mortality at 5000 mg/kg body weight which is the highest possible recommended dose for the toxicity test [15], a confirmatory test was carried out using two animals to validate the test dose. For any dose with signs of toxicity or mortality at any stage of the test, two animals were used to confirm the dose. The formula for calculating the LD_50_ after the toxicity test is given as follows:

(1) $$\begin{array}{c} {\text{LD}}_{50}=\frac{\left(D_{0}+D_{1}\right)}{2}, \end{array}$$

where *D*_0_ is the highest dose of test sample that gave no mortality or showed any sign of toxicity and *D*_1_ is the lowest dose of test sample that gave no mortality or showed any sign of toxicity.

#### 2.2.2. Experimental Rats

Thirty albino Wistar rats were purchased from the animal house of the College of Medical Sciences, University of Calabar, Calabar, Cross River State, Nigeria. The rats (6–8 weeks of age at the time) of average body weight 300 g were acclimatized for 3 weeks. The rats were allowed free access to water and food (growers mesh feed) under standard laboratory conditions of temperature, light, ventilation, and relative humidity.

#### 2.2.3. Induction of Anaemia

Phenylhydrazine chlorhydrate was used to induce haemolytic anaemia in the rats. DMSO solution was used to dissolve phenylhydrazine to one-tenth in distilled water. The solution was then administered intraperitoneally (IP) to the rats at a dose of 40 mg/kg of body weight/day for two days (D0 and D1).

#### 2.2.4. Experimental Protocol

At day 0, rats were randomly classified into the following 5 groups (6 rats per group): normal control group, phenylhydrazine (PHZ) group, PHZ + Astymin group, PHZ + extract (low dose) group, and PHZ + extract (high dose) group. Group 1 rats were not anaemic and served as control while the rats of other groups were anaemic. Group 3 rats were treated with Astymin. Groups 4 and 5 were treated with the extract 600 mg/kg of b.wt/day and 1000 mg/kg of b.wt/day, respectively, from D2 to D8. The extract and Astymin were administered by gavage using a gastric tube. Astymin is a blood tonic commonly prescribed for anaemic patients, in order to boost red blood cells production. The detail of the protocol is shown in Table 2.

### 2.3. Blood Sample Collection

Under chloroform anaesthesia, 2 rats from each group were dissected on days 3, 5, and 8. They were first weighed and then anaesthetized by placing them in a closed jar containing cotton wool soaked with 5 ml of chloroform anaesthesia. They were then dissected using surgical scissors to expose the heart; two blood samples were immediately withdrawn from the vena cava of each rat via cardiac puncture. The first sample was collected into a vial containing disodium salt of ethylene diamine tetra acetic acid (EDTA) anticoagulant. The second blood sample was collected into a plain sample vial without any anticoagulant.

### 2.4. Blood Tests

Approximately 5 ml of blood samples was collected in both EDTA and plain sample vials. Blood samples collected were used in the determination of haematological indices and liver enzymes.

- Estimation of haematological indices: Haematological parameters such as haemoglobin (Hb), red blood cell count (RBC), mean corpuscular volume (MCV), and mean corpuscular haemoglobin concentration (MCHC) and number of platelets were assessed by the standard haematological measurements using an automatic haematological assay analyser (Beckman Coulter, USA).
- Determination of liver enzymes: Blood serum enzyme levels of alanine aminotransferase (ALT), aspartate aminotransferase (AST), and alkaline phosphate (ALP) were determined using standard laboratory procedure at the University of Calabar Teaching Hospital Laboratory, chemical pathology unit.

### 2.5. Ethical Approval

Ethical clearance for this study was applied for, and duly obtained from the Faculty Animal Research Ethics Committee (FAREC-FBMS) of the Faculty of Basic Medical Sciences, College of Medicine, University of Calabar with reference number: FAREC/GP/008/21. All ethical procedures/rules were followed.

### 2.6. Statistical Analysis

The results were expressed as the mean ± standard error of mean (SEM) which were calculated from duplicate laboratory test values. Graphs were plotted using GraphPad Software. Differences among each group were investigated using one-way analysis of variance (ANOVA) with post hoc test for least significant difference (LSD) assuming equal variances using the IBM SPSS statistic software version 22 (SPSS: Statistical Package for Social Sciences). Significant difference was accepted at *p*  <  0.05.

## 3. Results

### 3.1. Acute Oral Toxicity Test Result

From acute oral toxicity tests carried out on mice in a bid to determine the LD_50_ of the extract using Lorke's method, results obtained (Table 3) showed no mortality within 24 hours after treatment with the extract with doses of up to 5000 mg/kg b.wt. No death was recorded among all the dose groups throughout the experimental period, thus being indicative of LD_50_ values of up to 5000 mg/kg b.wt and above.

### 3.2. Haematological and Liver Enzymes Analysis Results

From Table 4, a significant difference is observed in the haemoglobin concentration when comparing G2 to G1. With G2 having a lower Hb concentration in comparison to the control group (G1), a significant difference was also found between G2 and G3, with G2 maintaining a lower Hb concentration than G3. While G4 and G5 showed a statistically significant decrease in haematological parameters such as WBC, PCV, MCV, MCH, and MCHC, a drop is further observed when comparing G2 to G1. An increase in WBC, MCV, and MHC levels of G3 is noticed when compared to G2. Also, a significant decrease in WBC in both G4 and G5 is observed when compared to those of G3. An accompanying drop in PCV and MCV levels of G4 and G5 occurs as compared to those of the control group, G1. MCV values of G4 were also found to statistically differ from those of G2 and G3. While the MCH of G4 differed from G1 and G3, both MCH and MCHC of G5 differ significantly from G1, G2, and G3. For enzyme parameters, AST, ALT, and ALP showed a significant increase when comparing G2 to G1. A contrary significant decrease in AST, ALT, and ALP levels is noticed when comparing G3 to G2 with a marked difference (increase) in AST, ALT, and ALP levels in G4 and G5 when compared to G1 and G3. However, a significant difference in ALT and ALP levels is noticed between G5 and G2.

From Table 5, showing the result for day 5 of testing, a significant difference is observed in the PCV and MCV when comparing G2 and G3 to G1. A significant difference, however, still exists among these parameters when compared between the two groups, i.e., G3 and G2. A significant decrease in Hb level is observed when comparing G2 to G1 while G3 Hb level is significantly higher than G2. Significantly elevated levels of MCH and MCHC in G3 were noticed when compared to G1 and G2. Furthermore, a significant difference in Hb, PCV, and MCV levels in G4 is observed compared to G2 and G3. A significant difference in MCH and MCHC in G3 was noticed when compared to G1 and G2. Further difference in Hb, PCV, and MCV levels in G4 is observed compared to G2 and G3. A significant difference in MCH and MCHC levels of G4 occurs when compared to G1 while the MCHC level of G4 differs significantly from G3. In G5, RBC, Hb, PCV, and MCV levels significantly differ from both G1 and G3 while MCH and MCHC levels of G5 differ only from G3. For biochemical parameters, such as AST, ALT, and ALP, G2 levels are significantly higher when compared to G1 and G3. G4, AST, ALT, and ALP levels are also significantly lower compared to G2 while those of G5 tend to differ significantly from those of G1, G2, and G3.

**Figure 4:**
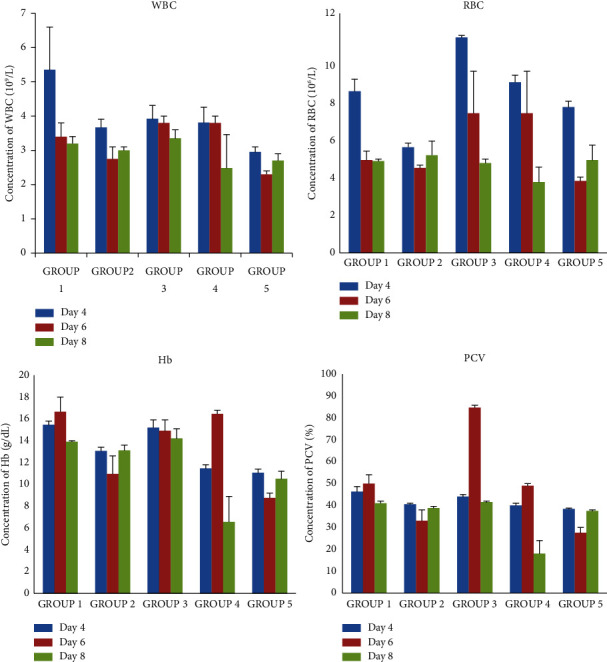
Changes in haematological indices on days 4, 6, and 8 of treatment. Bar 1 (blue): day 4, bar 2 (red): day 6, and bar 3 (green): day 8.

**Figure 5:**
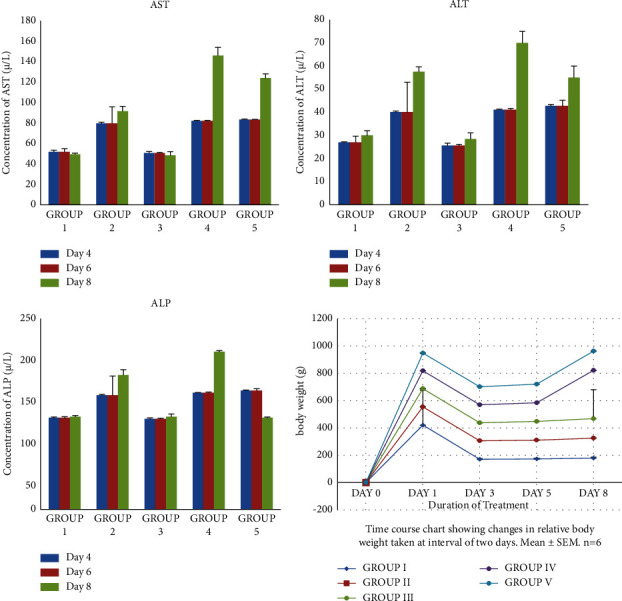
Changes in serum liver enzyme levels and relative body weight on days 4, 6, and 8. Bar 1 (blue): day 4, bar 2 (red): day 6, and bar 3 (green): day 8.

On day 8 of testing, Table 6 shows there was no significant difference in haematological parameters when comparing among the three groups: G1, G2, and G3. However, Hb, PCV, and MCH showed statistically significant differences when comparing their levels in G4 to those of G1, G2, and G3. Meanwhile in G2, AST, ALT, and ALP levels showed a significant increase when compared to those of G1 and G3. AST, ALT, and ALP levels of both G4 and G5 also showed significant increase compared to those of G1, G2, and G3. Figures 4 and 5 also give a pictorial representation of the haematological indices and liver enzymes at days 4, 6, and 8 after the extract administration.

## 4. Discussion

Similar to previous research results, values obtained on day 3 testing (Table 1), G2 which was administered phenylhydrazine (PHZ) showed a significant decrease in the haemoglobin (Hb) level compared to the control group G1. This decrease could be explained by the mechanism of action of PHZ, which induces haemolytic anaemia via the destruction of the red blood cells (RBCs) by oxidative stress which causes alterations to the RBCs proteins [3, 16]. The destruction of RBCs led to a concomitant release of the liver enzymes ALT, AST, and ALP into the blood serum, leading to a spike in the serum levels of these enzymes as observed from Table 1. However, these effects were effectively reversed in the group (G3) given the standard drug Astymin. The reversal of the debilitating effects of haemolytic anaemia and accompanying elevated liver enzyme levels is most likely due to the ability of Astymin to improve erythropoiesis, promote tissue repair, improve haemoglobin synthesis, protect the liver, and stimulate protein manufacture in the body [17–19].

In G4 and G5, fed with the low and high doses of the extract respectively, a significant decrease in Hb levels is observed with an accompanying increase in enzyme levels to values even higher than those of G2. The findings reported here suggest an inability of the fruit mesocarp extract to improve anaemic conditions.

From day 5 of testing (Table 2), the trend in decrease of Hb levels and elevation of liver enzyme levels in G2 is sustained. A possible explanation for this has been previously stated, which is due to the ongoing destruction of the RBCs [3, 16]. Additionally, G3 maintained healthy Hb and enzyme levels, thus lending credence to the potency of Astymin as a blood tonic and haematinic agent. In G4 and G5, an increase and decrease in Hb levels, respectively, are observed. This seems to be out of place with readings previously obtained in Table 1 where there was a uniform drop in Hb levels, and readings subsequently obtained in Table 3, where the Hb levels still fell significantly below the average healthy levels compared to the control (G1) and treated groups (G3). This inconsistency may be attributed to a myriad of reasons ranging from factors such as differences in the weights of the rats to physiological variations which may inevitably cause some of the rats to respond in a way different from the experimentally anticipated or expected outcome.

In Table 3 showing results for the final day of testing, a notable increase in Hb levels of G2 is noticed, having almost the same levels as those of G1. This phenomenon can readily be explained due to the regenerative ability of the RBCs which enables it to regenerate/repair itself after the agent which caused the haemolysis has been removed. Thus, it is most likely that this ability is what may have caused the Hb level to climb back up to normal levels after the considerable time lapse since the administration of PHZ. However, in towing this line, the interesting question may arise as to why it is not the same case in G4 and G5, which also had time to recuperate from the damaging effects of haemolytic anaemia. A possible explanation for this taking into consideration the fact that, while G2 was administered PHZ only and left to take the natural course of progression, G4 and G5 were constantly fed the extracts at low and high doses, respectively. Therefore, from the results obtained so far, it could be inferred that the fruit mesocarp may not only be ineffective in ameliorating or improving anaemic conditions due to the absence of key minerals and vitamins necessary to mediate this action but might also tend to worsen the case in a diseased condition. These findings seem to be in keeping with the research of Eseyin et al. [13], where it was suggested that the fruit extract may be unsafe for consumption due to its damaging effects on the rat liver and bones.

## 5. Conclusion

The aqueous extract of the fruit mesocarp of fluted pumpkin (*Telfairia occidentalis*) was discovered to be nontoxic at an acute level. Meanwhile, its efficacy as a haematinic agent in reversing PHZ-induced haemolytic anaemia and hepatoprotective ability is not proven and may on the contrary even pose a detrimental effect to health when ingested at high doses over a long period of time. Consequently, the leaves of the fluted pumpkin and not the fruit mesocarp may be used to boost blood levels until otherwise established.

## Figures and Tables

**Table 1:** Toxicity test protocol.

Stage	Group 1 (mg/kg b.wt)	Group 2 (mg/kg b.wt)	Group 3 (mg/kg b.wt)	Group 4 (mg/kg b.wt)
1	50	200	400	800
2	1000	1500	2000	
3	3000	4000	5000	

**Table 2:** Detail of the experimental protocol and design.

Group	Description	Procedure
1	Nonanaemic control group containing six (6) rats	Rats in this group were solely given distilled water with feed from D0 to D8
2	Anaemic control group containing six (6) rats	Rats were given phenylhydrazine at 40 mg/kg b.wt for two days (D0 and D1) and distilled water with feed from D2 to D8
3	Control reference group containing six (6) rats	Rats were given phenyl hydrazine at 40 mg/kg b.wt for two days (D0 and D1) and 1 ml/kg b.wt of Astymin from D2 to D8
4	Anaemic test group containing six (6) rats given low dose of extract	Rats were given phenylhydrazine at 40 mg/kg b.wt for two days (D0 and D1) in order to induce anaemia. They were then treated with 600 mg/kg b.wt of the *Telfairia occidentalis* fruit mesocarp aqueous extract from D2 to D8
5	Anaemic test group containing six (6) rats given high dose of the extract	Rats were given phenylhydrazine at 40 mg/kg b.wt for two days (D0 and D1) in order to induce anaemia. They were then treated with 1000 mg/kg/b.wt of the *Telfairia occidentalis* fruit mesocarp aqueous extract from D2 to D8

D0 = day the experiment was started; D1 = first day after the start of the experiment; D2 = second day after the start of the experiment; D8 = eighth day after the start of the experiment (also the last day of the experiment).

**Table 3:** Acute toxicity effect of aqueous extract of fruit mesocarp of *Telfairia occidentalis* administered orally to albino mice.

Experimental stage	Dose (mg/kg b.wt)	Number of dead mice after 24 hrs	Treated mice after 24 hours
Stage 1	50	0/1	0/1
	200	0/1	0/1
	400	0/1	0/1
	800	0/1	0/1

Stage 2	1000	0/1	0/1
	1500	0/1	0/1
	2000	0/1	0/1

Stage 3	3000	0/1	0/1
	4000	0/1	0/1
	5000	0/1	0/1

Confirmatory test	5000	0/2	0/2

Experiment was conducted in four stages; each dose group of stages 1–3 made up of 1 mouse for each dose while the confirmatory test consisted of 2 mice.

**Table 4:** Haematological indices and liver enzymes for the various rat groups at day 3.

Rat groups	RBC (10^6^/L)	WBC (10^9^/L)	Hb (g/dL)	PCV (%)	MCV (fL)	MCH (pg)	MCHC (g/dL)	AST (*μ*/L)	ALT (*μ*/L)	ALP (*μ*/L)
G1	5.35 ± 1.25	8.10 ± 0.60^#a^	15.45 ± 0.35^#^	46.30 ± 2.30^#^	85.85 ± 0.65^#^	24.70 ± 0.60^#^	43.55 ± 0.55^#^	51.90 ± 1.60^#^	26.95 ± 0.25^*∗*^^#^	120.00 ± 1.00^#^
G2	3.67 ± 0.24	5.30 ± 0.20^*∗*^^a^	13.05 ± 0.35^*∗*^^a^	40.50 ± 0.50^*∗*^	81.25 ± 0.95^*∗*^^a^	33.50 ± 0.40^*∗*^^a^	40.90 ± 0.60^*∗*^^a^	79.70 ± 1.10^*∗*^^a^	40.10 ± 0.40^a^	149.60 ± 1.00^*∗*^^a^
G3	3.92 ± 0.39	10.80 ± 0.10^*∗*^^#^	15.20 ± 0.70^#^	44.00 ± 1.00	85.65 ± 1.05^#^	25.75 ± 0.45^#^	44.00 ± 1.30^#^	50.75 ± 1.35^#^	25.55 ± 1.05^#^	118.50 ± 1.50^#^
G4	3.81 ± 0.45	8.55 ± 0.35^#a^	11.45 ± 0.35^*∗*^^#a^	40.00 ± 1.00^*∗*^	79.05 ± 0.85^*∗*^^#a^	33.70 ± 0.10^*∗*^^a^	41.75 ± 0.15	82.20 ± 0.40^*∗*^^a^	41.10 ± 0.20^*∗*^^a^	152.80 ± 0.20^*∗*^^a^
G5	2.95 ± 0.15	7.30 ± 0.30^#a^	11.05 ± 0.35^*∗*^^#a^	38.40 ± 0.40^*∗*^^a^	74.75 ± 0.55^*∗*^	30.75 ± 0.75^*∗*^^#a^	37.95 ± 0.25^*∗*^^#a^	83.55 ± 0.35^*∗*^^a^	42.70 ± 0.60^*∗*^^#a^	155.70 ± 0.70^*∗*^^#a^

Values are expressed as mean ± SEM. ^*∗*^Significantly different from G1 at *p*  <  0.05; ^#^significantly different from G2 at *p*  <  0.05; ^a^significantly different from G3 at <0.05 where, G1 = group 1 (rats given distilled water and feed only), G2 = group 2 (rats induced with phenylhydrazine and left untreated), G3 = group 3 (rats induced with phenylhydrazine and treated with Astymin), G4 = group 4 (rats induced with phenylhydrazine and given low dose of the extract), and G5 = group 5 (rats induced with phenylhydrazine and given high dose of the extract).

**Table 5:** Haematological indices and liver enzymes of the various rat groups at day 5.

Rat groups	RBC (10^6^/L)	WBC (10^9^/L)	Hb (g/dL)	PCV (%)	MCV (fL)	MCH (pg)	MCHC (g/dL)	AST (*μ*/L)	ALT (*μ*/L)	ALP (*μ*/L)
G1	3.40 ± 0.40	4.65 ± 0.45	16.65 ± 1.35^#a^	50.00 ± 4.00^#a^	82.90 ± 1.40^#a^	27.55 ± 2.55^a^	42.60 ± 1.60^a^	52.55 ± 3.05^#^	25.65 ± 2.65^#^	118.50 ± 1.50^#^
G2	2.75 ± 0.35^a^	4.25 ± 0.15	10.95 ± 1.65^*∗*^^a^	33.00 ± 5.00^*∗*^^a^	76.05 ± 3.35^*∗*^^a^	29.05 ± 3.05^a^	34.70 ± 4.90^a^	104.55 ± 16.15^*∗*^^a^	55.10 ± 12.90^*∗*^^a^	176.95 ± 25.05^*∗*^^a^
G3	3.80 ± 0.20^#^	7.00 ± 2.10	14.90 ± 1.00^#^	84.70 ± 1.10^*∗*^^#^	25.55 ± 1.05^*∗*^^#^	45.10 ± 0.40^*∗*^^#^	54.05 ± 0.45^*∗*^^#^	54.05 ± 0.45^#^	26.25 ± 0.55^#^	117.00 ± 1.00^#^
G4	3.80 ± 0.20^#^	7.00 ± 2.10	16.45 ± 0.35^#a^	49.00 ± 1.00^#a^	84.70 ± 1.10^#a^	25.55 ± 1.05^a^	44.9 ± 0.25^#a^	54.05 ± 0.45^#^	26.25 ± 0.55^#^	117.00 ± 1.00^#^
G5	2.30 ± 0.10^*∗*^^a^	3.60 ± 0.20	8.75 ± 0.45^*∗*^^a^	27.50 ± 2.50^*∗*^	75.70 ± 1.10^*∗*^^a^	25.50 ± 1.50^a^	34.80 ± 1.60^a^	120.00 ± 0.00^*∗*^^#a^	65.50 ± 2.50^*∗*^^a^	127.50 ± 2.50^#^

Values are expressed as mean ± SEM. ^*∗*^Significantly different from G1 at *p*  <  0.05. ^#^Significantly different from G2 at *p*  <  0.05. ^a^Significantly different from G3 at *p*  <  0.05.

**Table 6:** Haematological indices and liver enzymes for the various rat groups at day 8.

Rat groups	RBC (10^6^/L)	WBC (10^9^/L)	Hb (g/dL)	PCV (%)	MCV (fL)	MCH (pg)	MCHC (g/dL)	AST (*μ*/L)	ALT (*μ*/L)	ALP (*μ*/L)
G1	3.20 ± 0.20	4.60 ± 0.10	13.90 ± 0.10	41.00 ± 1.00	75.60 ± 2.00	31.90 ± 1.10	34.70 ± 0.40	49.35 ± 1.15^#a^	29.95 ± 2.05^#^	121.50 ± 1.50^#^
G2	3.00 ± 0.10	4.90 ± 0.70	13.10 ± 0.50	38.80 ± 0.80	74.05 ± 1.65	30.10 ± 0.30	31.15 ± 1.15	91.65 ± 4.65^*∗*^^a^	57.50 ± 2.10^*∗*^^a^	176.00 ± 7.00^*∗*^^a^
G3	3.35 ± 0.25	4.50 ± 0.20	14.20 ± 0.90	41.50 ± 0.50	75.85 ± 2.45	31.40 ± 3.20	34.35 ± 2.55	48.40 ± 3.60^#^	28.40 ± 2.70^#^	121.50 ± 3.50^#^
G4	2.48 ± 0.98	3.55 ± 0.75	6.55 ± 2.35^*∗*^^#a^	18.00 ± 6.00^*∗*^^#a^	74.00 ± 3.20	21.85 ± 1.75^*∗*^^#a^	32.80 ± 3.00	146.00 ± 8.00^#^^*∗*^^a^	70.00 ± 5.00^*∗*^^a^	206.50 ± 1.50^*∗*^^#a^
G5	2.70 ± 0.20	4.65 ± 0.75	10.50^*∗*^^#a^ ± 0.70	37.50 ± 0.50	77.25 ± 0.85	30.05 ± 0.85	38.20 ± 0.20	124.00 ± 4.00^*∗*^^#a^	55.00 ± 5.00^*∗*^^a^	120.00 ± 1.00^#^

Values are expressed as mean ± SEM. ^*∗*^Significantly different from G1 at *p*  <  0.05. ^#^Significantly different from G2 at *p*  <  0.05. ^a^Significantly different from G3 at *p*  <  0.05.

## Data Availability

The analytical data used to support the findings of this study are included within the article.
